# As low as reasonably practicable (ALARP): a moral model for clinical risk management in the setting of technology dependence

**DOI:** 10.1136/jme-2023-109111

**Published:** 2023-12-19

**Authors:** Helen Lynne Turnham, Sarah-Jane Bowen, Sitara Ramdas, Andrew Smith, Dominic Wilkinson, Emily Harrop

**Affiliations:** 1 Oxford University Hospitals NHS Foundation Trust, Oxford, UK; 2 Royal Aeronautical Society, London, UK; 3 Oxford Uehiro Centre for Practical Ethics, University of Oxford, Oxford, UK; 4 Helen and Douglas House, Oxford, UK

**Keywords:** Child, Decision Making, Ethics- Medical, Parental Consent

## Abstract

Children dependent on life-prolonging medical technology are often subject to a constant background risk of sudden death or catastrophic complications. Such children can be cared for in hospital, in an intensive care environment with highly trained nurses and doctors able to deliver specialised, life-saving care immediately. However, remaining in hospital, when life expectancy is limited, can considered to be a harm in of itself. Discharge home offers the possibility for an improved quality of life for the child and their family but comes with significant medical risks.

When making decisions for children, two ethical models predominate, the promotion of the child’s best interests or the avoidance of harm. However, in some circumstances, particularly for children with life-limiting and/or life-threatening illness, all options may be associated with risk. There are no good options, only potentially harmful choices.

In this paper, we explore decisions made by one family in such circumstances. We describe a model adopted from risk management programmes beyond medicine, which offers a potential framework for identifying risks to the child that are morally permissible. Some risks and harms to a child, not ordinarily permitted, may be acceptable when undertaken in the pursuit of a specified desired good, so long as they are as low as reasonably practicable.

## Introduction

 Children who are dependent on medical technology for survival (technology-dependent children, TDC) are a small but increasing population whose medical care offers new ethical challenges for the teams supporting them, their families and society.

Children might be dependent on technology to breathe for them (ventilators) via face masks or tracheostomy^1^, to drain fluid build-up around their brain (ventriculoperitoneal shunt, VPS) or to provide nutrition (via tubes directly placed into the stomach or intestine or directly into veins), to clear toxins from blood (dialysis) or to support a failing heart (ventricular assist device). Some children are dependent on such technology for months or years as a bridge to definitive treatment or until they become more stable and support is longer required. For some children, dependence is lifelong, and despite the support of medical technology many are expected to live short lives. TDC are at high risk of serious life-threatening adverse events from underlying disease processes or related to the technology.^1 2^

There is a subgroup of TDC who may be able to survive for months or years, but for whom there is also a risk of sudden catastrophic deterioration. Children in this situation can be cared for relatively safely in hospitals, but often the necessary nursing skills require them to be in an intensive care environment. Outside this environment, there is a risk of sudden death. As such these children are acutely life threatened as well as having a life-limiting underlying disease. But living life in a hospital comes at considerable personal cost for the child and their family. Families and health professionals may wonder whether it may be better to be at home, even if that would be at increased risk, or whether the opposite may be the least harmful alternative.

Here, we present (with parental permission), a real case of a child and her family who faced challenging risky decisions. We include some reflections by the parents about the decisions they made, and the risks involved.

## Gracie

Before Gracie was born her family knew that she would have many problems. Gracie had a large defect spina bifida along with hydrocephalus and structural abnormality of the brain stem. This combination of problems was at the more severe end of the disease spectrum, causing her brain stem to be pulled from below.

As a newborn baby, Gracie underwent repair of her spinal defect and insertion of a VPS. She was discharged home, but after a short period was readmitted to hospital and needed invasive ventilation to support her breathing. Over time, it became clear that Gracie was dependent on technology for survival. This technology included a VPS and a tracheostomy with invasive long-term ventilation to overcome hypoventilation, difficulty clearing airway secretions and to maintain airway patency. With these measures, Gracie began to thrive, she gained weight and her development progressed. It was hoped that if Gracie could survive her early life she would continue to improve, and with time wean from invasive ventilation and could grow out of a need for tracheostomy. Gracie would be wheelchair users and at best, also likely to have some developmental differences, for example, experiencing difficulties with numeracy and social communication.

However, Gracie suffered from sudden profound episodes of low oxygen levels that were life-threatening. She was dependent on continuous oxygen saturations monitoring.

It would be many years before it was clear if Gracie would improve enough to be able to live without a tracheostomy. Some children with her combination of problems can live a long-life without a tracheostomy, some need lifelong tracheostomy, but many die in infancy or early childhood.

The choices open to Gracie and her family were to try to discharge to home, understanding that it was at risk of sudden death or to continue to live in intensive care for years, potentially her entire life.

## Ethical discussion

Children who are unable to make or communicate their own choices need surrogates to make decisions for them. The usual decision-makers are their parents or guardians. There are four basic moral reasons for parents having this key role. First, parents typically know their children best and are well placed to understand what the child would choose for themselves if they were able. Second, parents must act to promote their child’s interests, to do so requires rights including the right to make decisions including medical decisions. Third, parents share the consequences of the decisions made for their children and therefore, their own interests are affected by those decisions. Finally, there is intrinsic value in parenthood, and in giving parents a significant role in key decisions for their children.^3^

There are two ethical principles that health professionals often draw on when considering decisions for children, the promotion of best interests and the avoidance of harm.

### Best interests

High-risk decisions such as those faced by mum Chloe and the professionals caring for Gracie are made with reference to the child’s best interests, as per influential legal and ethical guidelines.^4 5^ These are most commonly appealed to when clinicians seek to over-rule the choices of parents.

There are recognised criticisms of best interests as first, there may not be a single answer as to what is in a child’s best interests. The perception of what is best may vary from parent to parent, professional to professional, circumstance to circumstance. Parents are usually best placed to know what is valued by their child. There might be disagreement as to what course of action would best promote the child’s interests. Sometimes such disagreements may be based on lack of understanding or knowledge. Yet, as in this case there is more than one reasonable conclusion.^6^ In Gracie’s case the clinicians seek to support decisions by parents that can be argued to not be in her best interests rather than over-rule the choice of the parent.

Second, narrow interpretations of best interests can fail to account for the position of the child in her family and other important social and cultural factors. Gracie’s story illustrates that these difficult decisions are potentially life changing for all the family. Best interest considerations do not have to be exclusive to the child with medical needs and can encompass wider contextual factors.^7^ In practice, decisions in the best interests of the child are usually made with consideration to the interests of the wider family, without reaching a threshold where interests compete or offer significant harm.

Third, appealing to best interests is arguably overly onerous and, may in practice, be impossible to achieve. Decisions made for children that are suboptimal may be good enough or simply, sufficient^8^ and are both morally acceptable and in a child’s interests to continue to pursue.

If we accept (as we ordinarily do) that parents may make decisions that are not literally in their child’s best interests, we then need a different standard to determine decisions that parents should not be allowed to make. Typically, this is set at the level at which decisions become significantly harmful.

### The harm threshold

In practice, parents and guardians are tolerated to make decisions for their children that are not in their interests, but do not pose a significant rise of serious harm or simply decisions that are sufficient.^8 9^

A related concept is Gillam’s^10^ ‘Zone of Parental Discretion’. The ZPD is the moral space between decisions that are harmful and those that are clearly beneficial including decisions with more than one morally acceptable outcome, for example, a decision to either embark on intensive care or to provide palliative care for an extremely premature newborn. In identical clinical scenarios, parents may permissibly make either choice.

For options that are outside the zone of parental discretion, it is usually thought that professionals should not offer, and parents should not choose treatment options that pose a significant risk of serious harm to their children.

### When harm is unavoidable

The challenge, as illustrated by Gracie’s case above, is that in some circumstances (and TDC may be particularly subject to this) all available options appear to pose a significant risk of harm.

Gracie was unable to live without the support of a ventilator and a VPS. Even with the tracheostomy and ventilator, Gracie would have sudden, unexpected periods of profound hypoxia (low level of oxygen in the blood) that threatened her life. Gracie had very fragile skin that often-developed open wounds, around her neck exposing her VPS to skin bacteria and these could travel to her brain causing meningitis. Often children with her combination of problems die in early childhood (or before birth), rarely some will grow out of the need for a tracheostomy or support of a ventilator; however, the chance of this longer more usual life was lower than the likelihood of her living a very short life.

Gracie was at an extremely high risk of dying suddenly; her life was acutely threatened as well as time limited. The safest place to care for Gracie was in intensive care where the professionals around her were able, in the main, to manage these episodes of hypoxia. Preventing sudden death would require Gracie to live her life in hospital, but treating these episodes of hypoxia would not guarantee that she would live a long-life.

As Chloe reflects (below), living in hospital comes at a very great cost to the child and the wider family. Families are divided, siblings suffer from deprivation of experiences, are cared for by friends and families for long periods. The needs of siblings are by necessity, often secondary to those of the child with significant medical needs. Families suffer from financial stress, loss of occupation, difficulty eating adequately, isolation and mental health crises. Children living in ICU have very limited lives. Time outside in natural light, time spent with families, opportunities for sensory development are limited. Sleep disturbance, long periods of boredom, frustration and discomfort are common.^11^

For children who have a prospect of recovery, the harms associated with living in hospital for long periods might be justified and might be reasonable for some families, although not for others. The decision clearly falls within a zone of parental discretion.

The clinicians caring for Gracie wished to support parents in choosing the option that attended to her best interests, but risked serious harm. Appealing to BI alone or deferring to a harm threshold does not answer this real-world challenge when there are no good options and clinicians can experience moral distress and fear moral blame. When offering management that has tangible harms, that is, the sudden, potentially preventable death of a child there are justifiable concerns about being criticised or there being professional consequences to allowing such high-risk children to be cared for at home.

**Figure 1:**
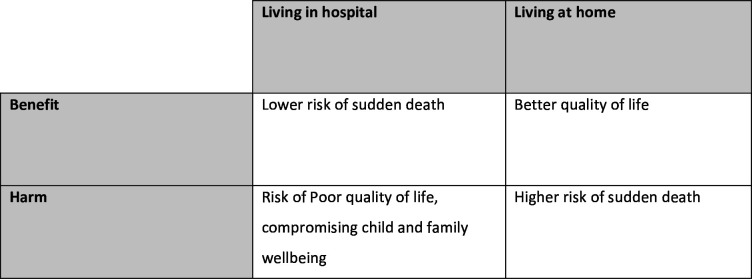
Matrix of benefits and harms.

To allow us to determine what is in the interests of Gracie, the clinical team needed to accept that there are actual harms that will be experienced as well as additional potential harms that might be experienced, whatever course is chosen (see figure 1).

## As low as reasonably practicable

**Figure 2:**
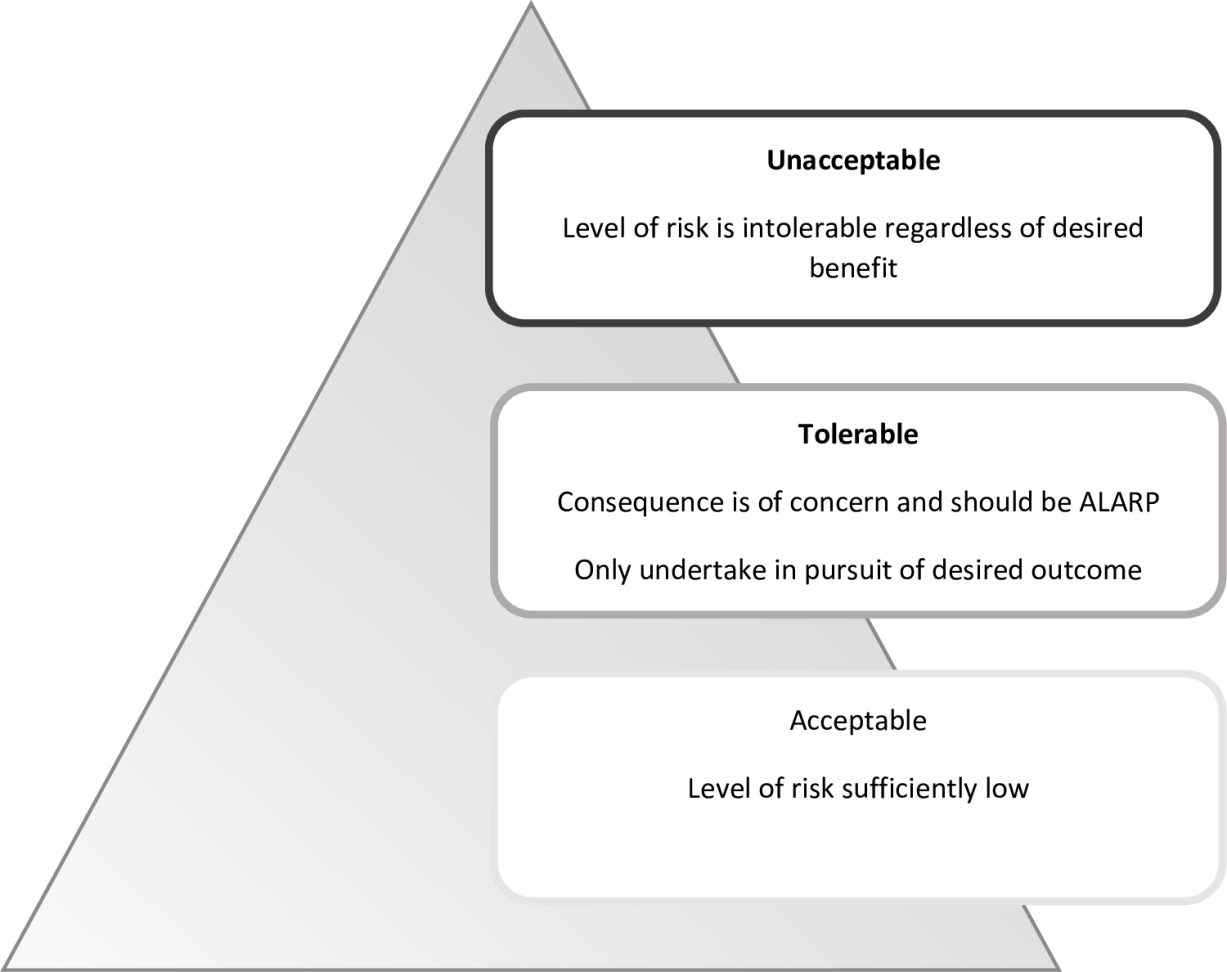
As low as reasonably practicable (ALARP) illustration.

In reflecting on cases like these, we adopted an idea common in risk management in other high-risk industries (see figure 2).

Most decisions made for children offer low risk of harm (eg, vaccinations) and are morally acceptable for doctors to offer and for parents to accept. At the other end of the spectrum are decisions that are unacceptable, that is, options that are extremely high risk or harmful regardless of a desired benefit. Between these extremes are options that have a potentially tolerable risk. It is important, however, that the risk undertaken is as low as reasonably practicable (ALARP).

The ALARP^12^ model acknowledges that there are moral predicaments when making decisions for children with no good choices, only harmful options. If these options were avoidable, there is no question that clinical teams should do so. Indeed, the potential harms are so great that it may be unethical to risk them if there were another way.

However, in circumstances like the ones we describe, adopting a high-risk strategy can be morally acceptable. Three conditions must be met:

1. Risks should be taken in the pursuit of a morally desirable outcome (eg, improving the quality of a child’s life, maximising time together as a family).
2. The risks should be mitigated or reduced to the lowest level practically achievable.
3. The overall balance of benefits and harms for the child should be positive.

Life for Gracie in hospital was limited, she could not easily play with her sister or stroke her dog. Visits by her large family were limited. Her family struggled emotionally, financially and practically. This was not a life that was enjoyable or of high quality although it was safer. Chloe and family wanted for Gracie a life that had meaning and joy, even if this might be shorter in length. The risk of death at home was also preferable to death in hospital without having benefited from time spent at home. For Gracie and her family pursuing a life at home, even a short period, was desirable over a longer life only lived in hospital.

## Gracie

Gracie’s family really wanted Gracie to live her life at home. Review of her case by the clinical ethics advisory group supported that this option would be ethical if the risks could be reduced to ALARP. A care package was set up with intensive nursing support. Finally, aged 16 months, she was able to be discharged, and spent a small amount of time at home. After just 1 week, she returned to hospital urgently with a pressure sore on her neck through which the VP shunt was visible. Sadly, Gracie developed a serious infection, deteriorated rapidly and died in hospital a few weeks later.

## Chloe’s story (Gracie’s mother)

“We would fight to do the same again”

Most of Gracie’s life was hard for us, we lived in hospital for most of her life and that was horrible, we had almost nothing, no home comforts and even managing to eat every day was hard. Paying the bills at home and trying to keep going in hospital was tough.

Waiting to get home was the worst time, finding and training carers who could look after Gracie took a long time, carers would let us down or were not able to deliver the care that Gracie needed. When we did have chances to go home, we were let down last minute and Ryan and I would both stay up all night caring for Gracie.

We spent a week at home with Gracie before she deteriorated; this was the happiest time of her and our lives. She loved being home and was mesmerised by our dog, Jack. Gracie played with her sister and having the wider family come and spend time with us was so special. We enjoy remembering when one of the hospital nurses came to visit and Gracie’s sister pushed her out of the front door saying, ‘Gracie is home, and she is ours’.

Even though Gracie died and only managed to have a week at home, this was our favourite times, and we would fight to do it the same all over again.

## Conclusion

In general, medical professionals should act in the best interest of children and not offer harmful intervention while parents should promote the BI of their children while not making harmful choices. However, in some circumstances, only potentially harmful options are available, despite appealing to the BI of the child. When there are tangible consequences, such as the death of a child, there is valid concern regarding criticism and professional consequences with moral blame for the parents and clinicians. Adopting an ALARP model to moral decision-making can aid families and medical teams around a child to make difficult but morally acceptable decisions.

## Data Availability

No data are available.

## Footnotes

Tracheostomy – formation of a stoma, a hole, in the front of the neck and insertion of a plastic tube directly into the trachea.
